# Perfluorooctane sulfonate (PFOS) and perfluorooctanoate (PFOA) acutely affect human α_1_β_2_γ_2L_ GABA_A_ receptor and spontaneous neuronal network function *in vitro*

**DOI:** 10.1038/s41598-020-62152-2

**Published:** 2020-03-24

**Authors:** Anke M. Tukker, Lianne M. S. Bouwman, Regina G. D. M. van Kleef, Hester S. Hendriks, Juliette Legler, Remco H. S. Westerink

**Affiliations:** Neurotoxicology Research Group, Toxicology Division, Institute for Risk Assessment Sciences (IRAS), Faculty of Veterinary Medicine, Utrecht University, P.O. Box 80.177, NL-3508 TD Utrecht, The Netherlands

**Keywords:** Cellular neuroscience, Excitability, Inhibition, Neurotransmitters

## Abstract

Concerns about the neurotoxic potential of polyfluoroalkyl substances (PFAS) such as perfluorooctane sulfonate (PFOS) and perfluorooctanoic acid (PFOA) increase, although their neurotoxic mechanisms of action remain debated. Considering the importance of the GABA_A_ receptor in neuronal function, we investigated acute effects of PFAS on this receptor and on spontaneous neuronal network activity. PFOS (Lowest Observed Effect Concentration (LOEC) 0.1 µM) and PFOA (LOEC 1 µM) inhibited the GABA-evoked current and acted as non-competitive human GABA_A_ receptor antagonists. Network activity of rat primary cortical cultures increased following exposure to PFOS (LOEC 100 µM). However, exposure of networks of human induced pluripotent stem cell (hiPSC)-derived neurons decreased neuronal activity. The higher sensitivity of the α_1_β_2_γ_2L_ GABA_A_ receptor for PFAS as compared to neuronal networks suggests that PFAS have additional mechanisms of action, or that compensatory mechanisms are at play. Differences between rodent and hiPSC-derived neuronal networks highlight the importance of proper model composition. LOECs for PFAS on GABA_A_ receptor and neuronal activity reported here are within or below the range found in blood levels of occupationally exposed humans. For PFOS, LOECs are even within the range found in human serum and plasma of the general population, suggesting a clear neurotoxic risk.

## Introduction

Perfluorooctane sulfonate (PFOS) and perfluorooctanoic acid (PFOA) are well-known perfluoroalkyl substances (PFAS) consisting of an eight-carbon chain in which hydrogen atoms have been substituted with fluorine. Their combined hydrophobic, hydrophilic, oleophobic and lipophobic properties make them ideal industrial surfactants for the manufacturing of consumer products, including paint, stain repellents, fire-fighting foams, and non-stick cookware coatings. The strong carbon-fluorine bond renders PFOS and PFOA highly persistent and studies have shown their presence in the environment, wildlife and even human blood (for reviews see^1,2^). By 2002, production of PFOS was phased out and production phase out of PFOA followed in 2006^3^. Recent studies indicate that these efforts may be responsible for a reduction in human blood levels in some areas, but the long half-lives of PFOS and PFOA result in slow elimination from environment and humans^4,5^.

Research has demonstrated that the (developing) nervous system is one of the most sensitive targets for PFOS and PFOA. In mice and rats exposed pre- and/or neonatally to PFOS or PFOA, increased motor activity, decreased habituation and deficits in spatial learning and memory abilities have been observed^6–11^. Developmental neurotoxicity has also been observed in other species, including chicken^12^ and zebrafish larvae^13,14^. However, epidemiological studies have been inconclusive on the risks of PFAS exposure on neurodevelopment. Some studies indicate an association between prenatal PFOS and/or PFOA exposure and an increased risk on congenital cerebral palsy^15^, neuro-behavioural development^16^ and visual motor abilities^17^, whereas others reported no association between PFOS and PFOA exposure on psychomotor, cognitive and neurobehavioural development^18^, IQ levels^19^ or increased risk on ADHD and childhood autism^20^. Notably, most early studies reported no or limited neurodevelopmental or neurological effects of PFAS exposure^16,18,21–23^, although recent epidemiological studies indicate an association between PFAS exposure and child behaviour^24,25^. It is important to keep in mind that exposure to PFAS in these studies is continuous and/or based on prolonged exposure paradigms.

The mechanism(s) underlying the observed neurotoxicity have been studied *in vitro* in neuronal cells. At (high) micromolar concentrations, PFOS and/or PFOA can affect a range of (pre)-synaptic processes, including reduction of cell viability^26–29^, altered neuronal differentiation^30^, increased formation of reactive oxygen species^27,28,31–34^, and increased intracellular calcium (Ca^2+^) concentrations^35,36^. In addition, PFOS and PFOA may also affect postsynaptic processes, including altered glutamate-activated currents and increase of potassium currents^37,38^. By inducing influx of Ca^2+^ through voltage-dependent calcium channels, PFOS may exhibit acute excitotoxic effects on synaptic function and chronically inhibit synaptogenesis in rat brain hippocampal neurons^39^. Although *in vivo* studies suggest the involvement of the cholinergic^9^ and glutamatergic system^40^ in PFOS and PFOA mediated neurotoxicity, additional studies on postsynaptic receptors and channels by PFOS or PFOA are lacking.

Previous studies have shown agonistic effects on the postsynaptic human GABA_A_ receptor by several persistent organic pollutants, including polychlorinated biphenyls and brominated flame retardants^41–44^. The GABA_A_ receptor is the main inhibitory neurotransmitter receptor in the central nervous system and is critical for brain development^45^, long-term potentiation and synaptic plasticity^46^, but also for functional neuronal signal transduction. Because the GABA_A_ receptor is the most important inhibitory receptor in the central nervous system, any effect of PFAS on this receptor may in turn affect neuronal signal transduction and spontaneous neuronal network activity.

In the present study we therefore used *Xenopus* oocytes that express the human α_1_β_2_γ_2L_ GABA_A_ receptor, which is the most abundant subunit combination^47^, to investigate the acute effects of PFOS and PFOA using the two-electrode voltage-clamp technique. Additionally, we investigated whether the effects of PFOS and PFOA on the α_1_β_2_γ_2L_ GABA_A_ receptor are reflected in the level of spontaneous neuronal network activity using micro-electrode array (MEA) recordings in rat primary cortical neuronal networks, the current gold standard for MEA assays. These experiments were followed by measurements in human iPSC-derived neuronal models to investigate whether they were similarly affected by PFAS exposure as the rodent model.

## Results

### Antagonistic effects of PFAS on human GABA_A_ receptor

Oocytes expressing the human α_1_, β_2_, γ_2L_ subunits were superfused with various concentrations of GABA to derive a concentration-response curve with an EC_20_, EC_50_ and Hill slope of 36 µM, 103 µM and 1.3, respectively (see Fig. S1 and in line with previous publications^48^). The expression and functionality of the γ_2L_ subunit was demonstrated by co-exposing GABA-responsive oocytes to a GABA-diazepam co-exposure (see Fig. S1).

**Figure 1:**
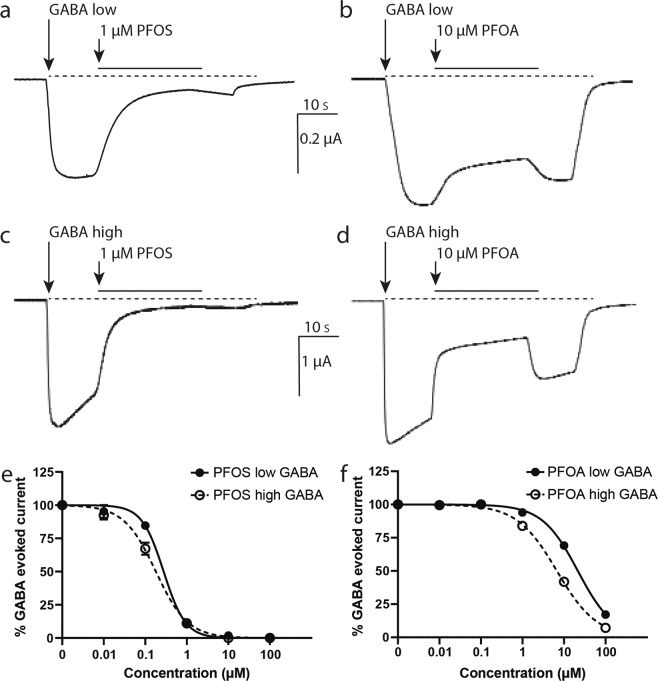
Antagonistic effects of PFOS and PFOA on the human GABA_A_ receptor. Example recordings of inhibition of GABA-evoked ion currents by co-application of PFOS (1 µM, **a**, **c**) or PFOA (10 µM, **b**, **d**) with GABA (at low and high effective GABA concentration, respectively). PFOS-induced inhibition is poorly reversible (**a**, **c**), whereas PFOA-induced inhibition is reversed within seconds (**b**, **d**). Scale bar applies to all traces. Concentration-response curves show the concentration-dependent inhibition of GABA-evoked responses by PFOS (**e**) and PFOA (**f**) on the human GAB_A_ receptor with at low (**e**,**f**; solid line) and high (**e,f**; dashed line) effective GABA concentrations. Inhibition is presented as percentage of the GABA-evoked response (mean ± SEM, *n* = 3–4 oocytes per concentration from N = 1–2 batches).

To investigate whether PFOS and PFOA are able to activate the human GABA_A_ receptor, GABA-responsive oocytes were superfused with saline containing 0.1–100 µM PFOS or PFOA. Neither PFOS nor PFOA induced any ion current, demonstrating that PFOS and PFOA by themselves are not agonists of the GABA_A_ receptor (data not shown). To determine possible partial agonistic or antagonistic effects, PFOS and PFOA were co-applied with a low concentration of GABA (~EC_20_) and the lowest observed effect concentrations (LOEC; defined as the lowest test concentration that is significantly different from the control) of PFOS and PFOA were calculated. At this low effective GABA concentration, both PFOS (LOEC 0.1 µM, IC_50_ 0.28 µM, CI 0.22–0.34 µM, see Fig. 1a,e and Table 1; F(1,21) = 10.2, *p* = 0.004) and PFOA (LOEC 10 µM, IC_50_ 22 µM, CI 19.28–24.21 µM, see Fig. 1b,f and Table 1; F(1,22) = 256, *p* = <0.0001) concentration-dependently inhibited the GABA-evoked ion current. These data demonstrate that both PFOS and PFOA exert antagonistic effects on the GABA_A_ receptor. Notably, as shown in Fig. 1a, the PFOS-induced inhibition of the GABA-evoked ion current is not readily reversible. In contrast, the PFOA-induced inhibition is reversed within seconds (Fig. 1b).

**Table 1:** Inhibition of GABA-evoked ion current by PFOS or PFOA.

	Concentration (µM)	Low effective GABA concentration		High effective GABA concentration	
		% Inhibition	*p-value*	% Inhibition	*p-value*
PFOS	0.01	4.8 ± 4.8 (4)	n.s	7.6 ± 3.3 (4)	n.s
	0.1	15 ± 1.8 (4)	0.002	33 ± 4.6 (4)	<0.0001
	1	90 ± 1.5 (3)	<0.0001	89 ± 0.9 (3)	<0.0001
	10	98 ± 1.2 (4)	<0.0001	100 ± 0 (3)	<0.0001
	100	100 ± 0 (4)	<0.0001	100 ± 0 (4)	<0.0001
PFOA	0.01	0 ± 0 (4)	n.s.	0.4 ± 0.5 (4)	n.s
	0.1	0 ± 0 (4)	n.s.	0 ± 0 (3)	n.s
	1	6 ± 0.4 (4)	n.s	16 ± 0.4 (4)	<0.0001
	10	31 ± 1.9 (4)	<0.0001	58 ± 1.5 (4)	<0.0001
	100	83 ± 2.7 (4)	<0.0001	93 ± 2.7 (4)	<0.0001

Mean percentage of inhibition of the current (± SEM (*n*)) induced during exposure to PFOS or PFOA. Inhibitions were compared to control and *p-*values are given; n.s.: non-significant.

Next, PFOS and PFOA were co-applied with a high concentration GABA (1 mM, EC_100_). PFOS induced a poorly reversible, concentration-dependent inhibition of the GABA-evoked ion current (LOEC 0.1 µM, IC_50_ 0.18 µM, CI 0.15–0.22 µM; F(1,20) = 10.4, *p* = 0.004; Fig. 1c,e and Table 1), whereas the concentration-dependent PFOA-induced inhibition is rapidly reversed (LOEC 1 µM, IC_50_ 6.7 µM, CI 5.96–7.59 µM; F(1,22) = 66.2, *p* = <0.0001; Fig. 1d,f and Table 1).

The combined data indicate that PFOS and PFOA are potent, non-competitive GABA_A_ receptor antagonists. As such, PFOS and PFOA may antagonize the inhibitory function of the CNS. To determine whether the effects of these PFAS also modulate neuronal network function, effects of PFOS and PFOA on spontaneous neuronal network activity were assessed.

### Effects of PFAS on rat cortical network activity

Recently, non-invasive measurements of neuronal activity using MEA recordings have shown that rat primary cortical cultures develop spontaneous network activity^49^, which can be modulated by a diverse range of chemicals^50–53^. Currently, rat primary cortical neurons are the gold standard for MEA recordings. At DIV9, network bursts throughout the well can be observed. Therefore, we exposed rat primary cortical cultures at DIV9–11 to PFOS or PFOA to assess effects on spike, burst and network burst related parameters.

**Figure 2:**
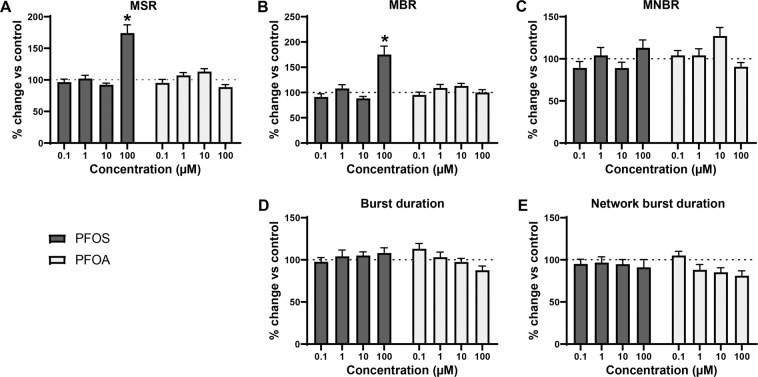
Modulation of spontaneous neuronal activity of rat primary cortical neurons exposed to PFOS (black) or PFOA (grey) following acute exposure. Effects on mean spike rate (MSR; **A**), mean burst rate (MBR; **B**), mean network burst rate (MNBR; **C**), burst duration (**D**) and network burst duration (**E**) are depicted as average in % change of control (solvent control set to 100%; dashed line) ± SEM from *n* = 18–20 wells and N = 4 plates. *Indicates *p* < 0.05.

Acute exposure to PFOS or PFOA affected mean spike rate (MSR; Fig. 2A), with statistically significant differences between the concentrations (Welch’s F(4, 44.7) = 9.76, *p* = <0.0001 for PFOS and Welch’s F(4, 46.5) = 3.5, *p* = 0.01 for PFOA). Following exposure to 100 µM PFOS, a marked increase in MSR could be observed (175 ± 13.1%; *p* = <0.0001). PFOS also increased the mean burst rate (MBR; Fig. 2B), with a statistically significant difference between concentrations (Welch’s F(4, 46.0) = 7.2, *p* = 0.0001). However, this increase was only significant compared to control following exposure to 100 µM PFOS (175 ± 16.7%; *p* = <0.0001). PFOS did not affect mean network burst rate (MNBR; Fig. 2C). PFOA only induced minor effects on spike and (network) burst rate. PFOA increased MNBR at 10 µM, but this increase was not significant. Neither PFOS nor PFOA affected burst duration (Fig. 2D), although there is a significant difference between groups for PFOA (Welch’s F(4, 45.8) = 3.81, *p* = 0.009). Both PFAS did not affect network burst duration (Fig. 2E), but for PFOA the one-way Welch ANOVA was significant (Welch’s F(4, 45.9.) = 3.67, *p* = 0.011). Effects observed at the highest test concentration are not a result of cytotoxicity (see Supplemental Data Fig. S2).

Overall, PFOS has a more profound effect on spontaneous neuronal network activity than PFOA. However, these data indicate that PFOS and PFOA induce only mild hyperexcitation in rat primary cortical cultures, which was unexpected considering the strong inhibition of human GABA_A_ receptors. This apparent discrepancy may be the result of inter-species differences. To investigate whether this is the case, human induced pluripotent stem cell (hiPSC)-derived neurons can be used. It is known that there is variability between different hiPSC-derived neuronal cell lines^54^. This could be the result of the generation protocol or the maturation stage in which the neurons were frozen. To take these potential differences into account, two commercially available hiPSC-derived neuronal cell models were exposed to PFOS or PFOA: the iCell Glutaneuron – iCell Astrocytes co-culture and the SynFire iNS co-culture.

### Effects of PFAS on spontaneous hiPSC network activity

**Figure 3:**
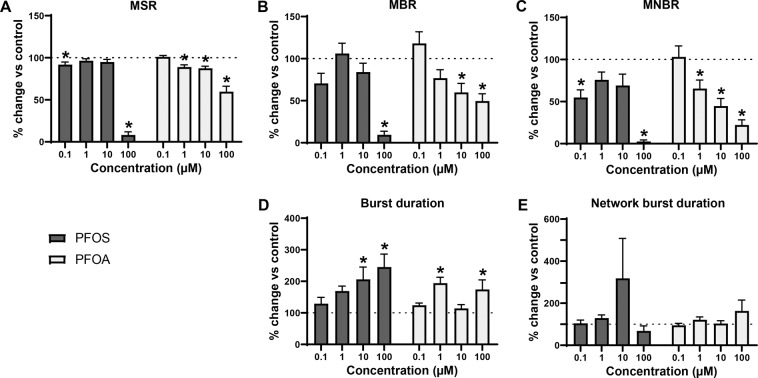
Modulation of spontaneous neuronal activity of iCell Glutaneuron – iCell Astrocytes co-culture exposed to PFOS (black) or PFOA (grey) following acute exposure. Effects on mean spike rate (MSR; **A**), mean burst rate (MBR; **B**), mean network burst rate (MNBR; **C**), burst duration (**D**) and network burst duration (**E**) are depicted as average in % change of control (solvent control set to 100%; dashed line) ± SEM from *n* = 3–17 wells and N = 3–5 plates. *Indicates *p* < 0.05.

**Figure 4:**
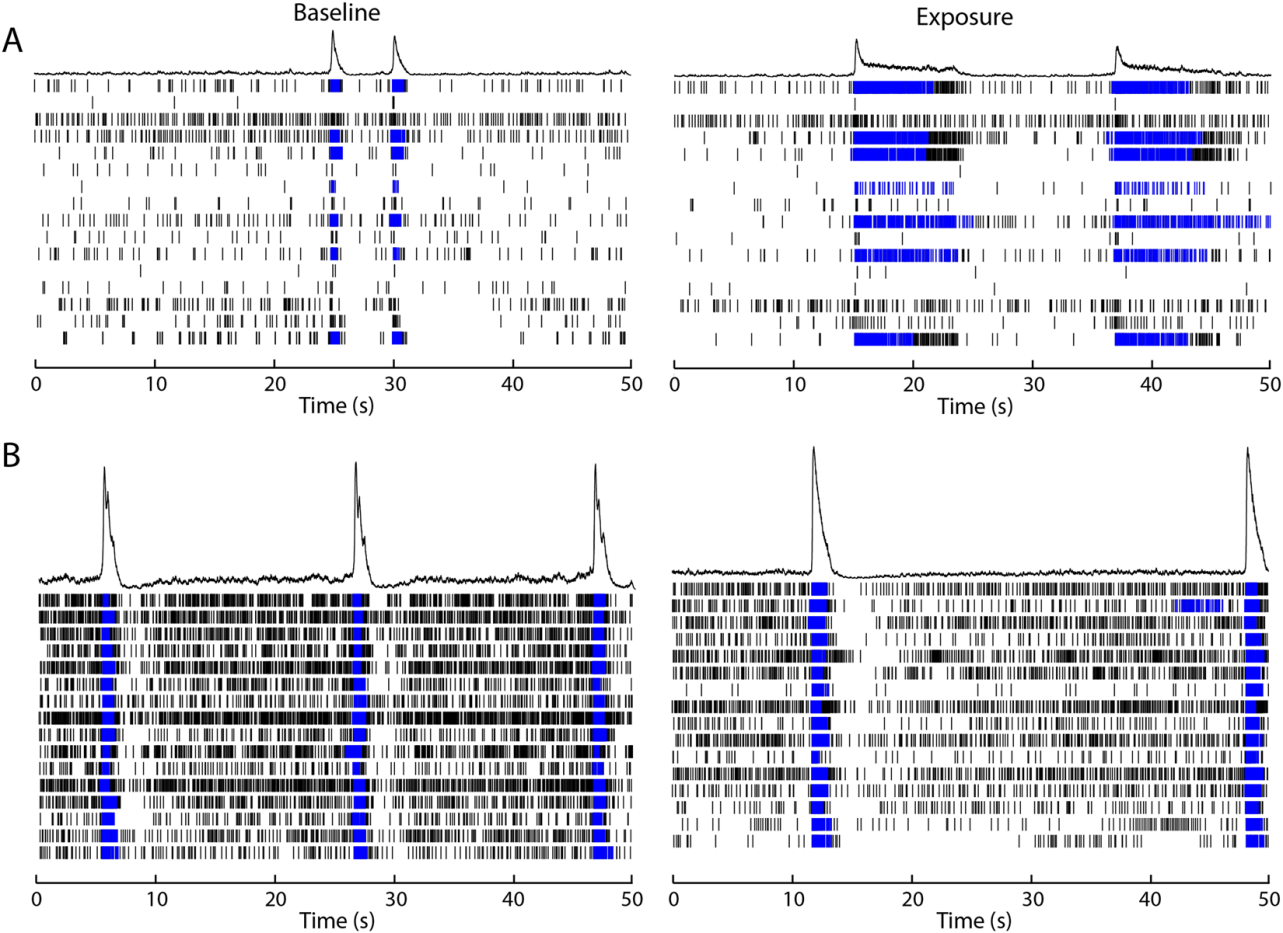
Spike raster plots illustrating the pattern of activity of a representative well of the iCell Glutaneuron – iCell Astrocytes co-culture before exposure (left) and the same well following exposure (right) to PFOS 10 µM (**A**) or PFOA 100 µM (**B**). Each row depicts one electrode in a well and each tick mark represents one spike in a 50 s interval. Spikes are depicted in black and bursts are depicted in blue.

Recent work in our laboratory has shown that hiPSC-derived neuronal co-cultures develop spontaneous activity and (network) bursting behaviour^55^. Acute exposure to PFOS (Welch’s F(4, 34.9) = 278, *p* = <0.0001) and PFOA (Welch’s F(4, 32.9) = 13, *p* = <0.0001) affected MSR in the iCell Glutaneuron – iCell Astrocytes co-culture (Fig. 3A)). PFOS significantly decreased the MSR at 0.1 µM (91.8 ± 3.18%; *p* = 0.04) and 100 µM (8.2 ± 3.71%; *p* = <0.0001). For PFOA, MSR was significantly decreased at 1 µM (88.9 ± 2.64; *p* = 0.02), 10 µM (87.4 ± 2.5%; *p* = 0.02) and 100 µM (59.7 ± 6.49; *p* < 0.0001) as compared to the control. Bursting was significantly affected following exposure to either PFAS (Welch’s F(4, 34.9) = 36.0, *p* < 0.0001 for PFOS and Welch’s F(4, 36.7) = 6.67, *p* = 0.0004; Fig. 3B). PFOS exposure significantly decreased MBR at 100 µM (9.43 ± 4.2%; *p* = <0.0001). For PFOA, MBR significantly decreased at 10 µM (59.8 ± 10.7%; *p* = 0.023) and 100 µM (49.5 ± 8.73%; *p* = 0.0035). MNBR was decreased following exposure to PFOS (Welch’s F(4, 30.1) = 51.0, *p* = <0.0001) and PFOA (Welch’s F(4, 35.4) = 15.6, *p* = <0.0001; Fig. 3C). The decrease was significant following exposure to PFOS at 0.1 µM (54.8 ± 9.1%; *p* = 0.002) and 100 µM (2.58 ± 1.9%; *p* < 0.0001) and for PFOA at 1 µM (65.4 ± 10.1%; *p* = 0.01), 10 µM (44.7 ± 9.03; *p* = 0.0005) and 100 µM (22.2 ± 6.11; *p* < 0.0001). Exposure of the iCell Glutaneuron – iCell Astrocytes co-culture to either of the PFAS increased burst duration (Welch’s F(4, 24.0) = 6.74, *p* = 0.0009 for PFOS and Welch’s F(4, 31.6) = 6.71, *p* = 0.0005 for PFOA; Fig. 3D). The increase in burst duration following exposure to PFOS was significant at 10 µM (206 ± 39.3; *p* = 0.002) and 100 µM (245 ± 41.3%; *p* = 0.0002). Following exposure to PFOA, the increase was significant at 1 µM (194 ± 18.9%; *p* = <0.0001) and 100 µM (174 ± 30.6; *p* = 0.01). There was no significant effect on network burst duration (Fig. 3E), despite an apparent increase following exposure to 10 µM PFOS. This change in activity pattern is further illustrated by spike raster plots that show the decrease in spiking and (network) bursting activity combined with prolonged (network) burst duration following exposure to 10 µM PFOS (Fig. 4A) or 100 µM PFOA (Fig. 4B).

Overall, effects are more pronounced following exposure to PFOS. However, PFOS and PFOA do not induce hyperexcitation in the iCell Glutaneuron – iCell Astrocytes co-culture model. Since the chemical sensitivity can differ between different hiPSC-derived neuronal cultures^56^, an additional hiPSC-derived cell model was tested, the SynFire iNS co-culture.

**Figure 5:**
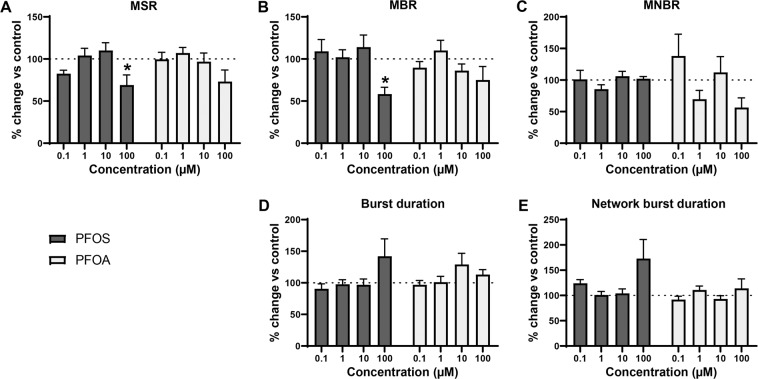
Modulation of spontaneous neuronal activity of SynFire iNS co-culture with PFOS (black) or PFOA (grey). Effects on mean spike rate (MSR; **A**), mean burst rate (MBR; **B**), mean network burst rate (MNBR; **C**), burst duration (**D**) and network burst duration (**E**) are depicted as average in % change of control (solvent control set to 100%; dashed line) ± SEM from *n* = 7–9 wells and N = 2 plates. *Indicates *p* < 0.05.

The SynFire iNS co-culture contains a higher ratio of astrocytes compared to the iCell Glutaneuron – iCell Astrocytes co-culture and although the ratio glutamatergic to GABAergic neurons is comparable, the total cell number is much higher thus the number of glutamatergic neurons is also higher in this culture^56^. Therefore, the SynFire iNS co-culture is more active and excitable. Exposure of SynFire iNS co-culture to PFOS affected the MSR (Welch’s F(4, 16.9) = 3.18, *p* = 0.04 for PFOS) with a significant decrease at 100 µM (69.1 ± 12; *p* = 0.04; Fig. 5A) as compared to control. Exposure to PFOA decreased the MSR at 100 µM, but this decrease was not significant. MBR was only significantly decreased by PFOS (Welch’s F(4, 18.2) = 5.24, *p* = 0.006) at 100 µM (58.3 ± 8.13; *p* = 0.04; Fig. 5B). Neither PFOS nor PFOA significantly disturbed MNBR (Fig. 5C). Burst duration and network burst duration were not significantly affected, although an increase could be observed following exposure to PFOS 100 µM (Fig. 5D,E).

### Comparison of PFAS effects in different cell models

LOECs for the activity metrics MSR, MBR and MNBR differ between the three different cell models (Table 2). The iCell Glutaneuron – iCell Astrocytes co-culture is the only model for which LOECs could be defined for PFOS and PFOA on all three metrics. This hiPSC-derived neuronal model is also the most sensitive of the three. The rat primary cortical neurons and SynFire iNS co-culture model show comparable LOECs, but respond differently to PFOS and PFOA exposure.

**Table 2:** LOECs of PFOS and PFOA for the different activity parameters (mean spike rate (MSR), mean burst rate (MBR) and mean network burst rate (MNBR)) on the different cell models. – Indicates no LOEC could be defined.

	MSR		MBR		MNBR	
	PFOS	PFOA	PFOS	PFOA	PFOS	PFOA
Rat primary cortical neurons	100 µM	—	100 µM	—	—	—
iCell Glutaneuron – iCell Astrocytes co-culture	0.1 µM	1 µM	100 µM	10 µM	0.1 µM	1 µM
SynFire iNS co-culture	100 µM	—	100 µM	—	—	—

**Figure 6:**
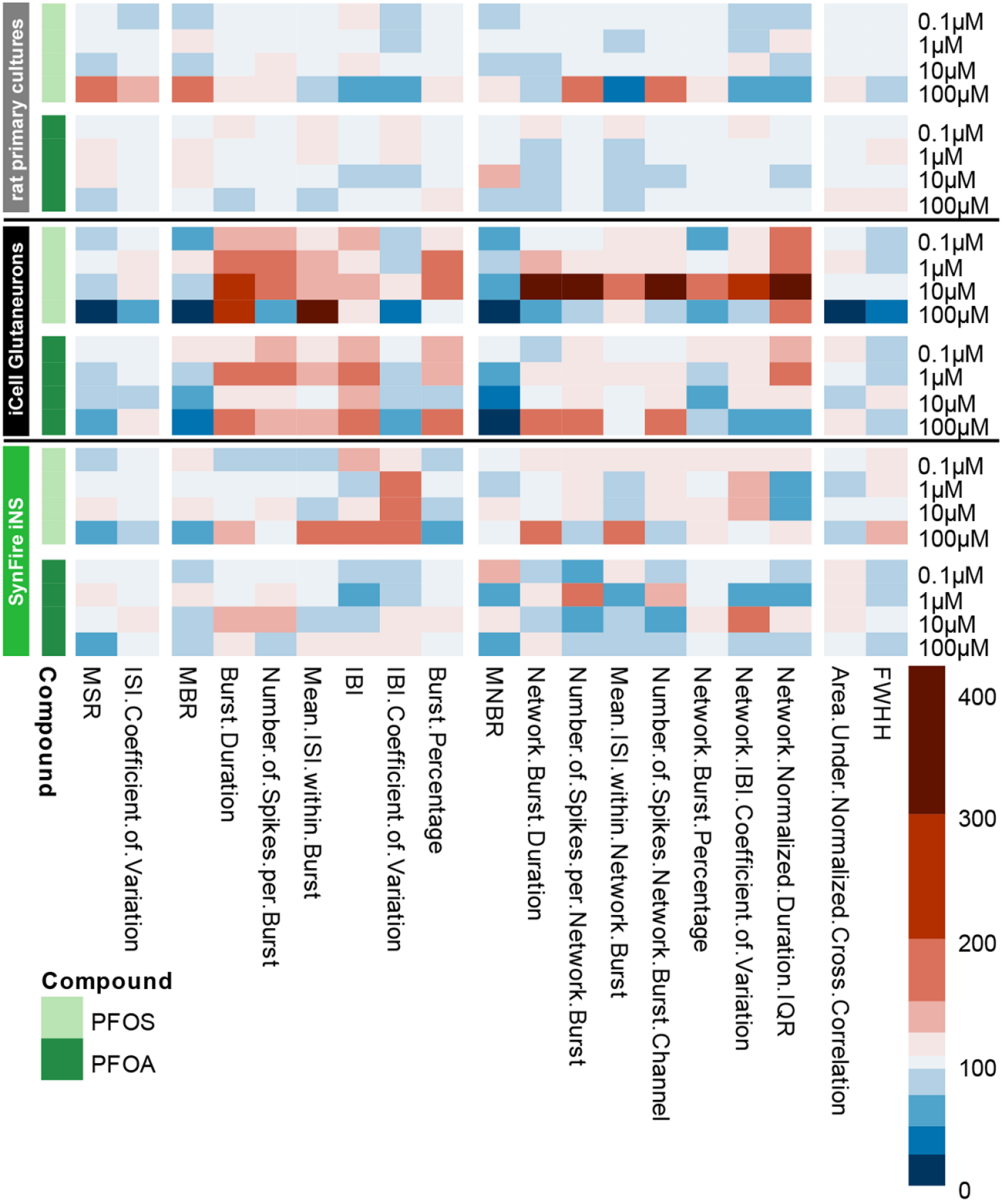
Heatmap of the effects of PFOS (light green) and PFOA (darker green) on selected metric parameters on rat primary cortical neurons (top; grey), iCell Glutaneuron – iCell Astrocytes (middle; black) and SynFire iNS co-culture (bottom; green). Colour scaling is based on the magnitude change in percentages relative to vehicle control based on *n* = 18–20 wells and N = 4 plates for rat primary cortical neurons; *n* = 3–17 wells and N = 3–5 plates for iCell Glutaneuron – iCell Astrocytes; *n* = 6–9 wells N = 2 plates for SynFire iNS co-culture.

To further illustrate the effect of PFOS and PFOA on the rat primary cortical neurons and the two hiPSC-derived neuronal models, we created a heatmap including 19 parameters that are descriptive for neuronal (network) activity (Fig. 6; Table 3). Although many changes (and some concentration-dependent changes) are visible in the heatmap and in Fig. 3, it should be noted that many of these changes do not reach statistical significance. Nevertheless, it is clear that the rat cortex is rather insensitive, though an increased activity can be observed following exposure to 100 µM. The iCell Glutaneuron – iCell Astrocytes co-culture model is the most sensitive model. This can be seen from the profound decrease in MSR, MBR and MNBR and the increase in burst duration. The SynFire iNS co-culture is just as (in)sensitive as the rat primary cortical culture (Figs. 2, 5 and 6), though with an increase, in contrast to a decrease, in activity following exposure to 100 µM. It is also apparent that PFOS has a stronger and more excitatory effect than PFOA. In all three models, synchronicity parameters are least affected. Overall, this highlights that there are differences between human and rodent models. It also indicates that there are differences between the human model systems. Overall the model system used can to a large degree determine the type and degree of the effect.

**Table 3:** Description of different metric parameters.

Metric parameter	Description
Mean spike rate (MSR)	Total number of spikes divided by recording time (Hz)
ISI coefficient of variation	Standard deviation ISI (time between spikes) divided by the mean ISI. Measure for spike regularity: 0 indicates perfect spike distribution, >1 signals bursting
Mean burst rate (MBR)	Total number of bursts divided by recording time (Hz)
Burst duration	Average time from the first spike in a burst till the last spike (s)
Number of spikes per burst	Average number of spikes occurring in a burst
Mean inter-spike interval (ISI) within burst	Mean inter-spike interval within a burst (s)
Inter-burst interval (IBI)	Time between the last spike of a burst and the first spike of a subsequent burst (s)
IBI coefficient of variation	Standard deviation of IBI divided by the mean IBI. Measure for burst regularity
Burst percentage	Percentage of total number of spikes occurring in a burst
Mean network burst rate (MNBR)	Total number of network bursts divided by recording time (Hz)
Network burst duration	Average time from the first spike till the last spike in a network burst (s)
Number of spikes per network burst	Average number of spikes occurring in a network burst
Mean ISI within network burst	Average of the mean ISIs within a network burst (s)
Number of spikes per network burst per channel	Average number of spikes across the network bursts in the network burst divided by the number of electrodes participating in the network burst
Network burst percentage	Percentage of total spikes occurring in a network burst
Network IBI coefficient of variation	Standard deviation of network IBI divided by the mean network IBI. Measure of network burst rhythmicity: value is small when bursts occur at regular interval and increases when bursts occur more sporadic
Network normalized duration IQR	Interquartile range of network bursts durations. Measure for network burst duration regularity: larger values indicate wide variation in duration.
Area under normalized cross-correlation	Area under inter-electrode cross-correlation normalized to the auto-correlations. The higher the value, the greater the synchronicity of the network
Full width at half height (FWHH) of normalized cross-correlation	Width at half height of the normalized cross-correlogram. Measure for network synchrony: the higher the value, the less synchronized the network is

Adapted from^56^.

## Discussion

PFOS and PFOA are omnipresent in the environment. *In vivo* and *in vitro* research have indicated the neurotoxic potential of both compounds^57^. However, the underlying mechanisms of action are still debated. In the present study we show for the first time that PFOS and PFOA exhibit concentration-dependent antagonistic effects on the human α_1_β_2_γ_2L_ GABA_A_ receptor at sub-micromolar concentrations (Fig. 1, Table 1). It is important to note that we tested only the most abundant α_1_β_2_γ_2L_ receptor subunit combination. As many other GABA_A_ receptor subtypes are present in the nervous system, PFAS may have different effects on these different receptor subtypes. The GABA-evoked current is rapidly restored once exposure to PFOA stops, but reversibility of the effect caused by PFOS is slow. This indicates that PFOA most likely has a lower receptor affinity than PFOS, which could be caused by the differences in head group. The reversibility could explain why at a high effective GABA concentration the LOEC of PFOA (1 µM) is tenfold higher than the LOEC of PFOS (0.1 µM). The observed inhibition of the GABA-evoked current occurs independently of the effective GABA concentration, implying that both PFAS are non-competitive GABA antagonists. This also indicates that neither PFOS nor PFOA binds to the GABA binding site located at the interface between the α and β subunit, but are more likely to bind to modulatory binding sites. A variety of compounds, including convulsants and barbiturates, target these modulatory binding sites^58^. However, when positive or negative allosteric modulators bind to modulatory binding sites, the effect of the compound usually decreases with an increasing concentration of GABA. This is in contrast with our results. The agonistic effects of PFOS and PFOA could therefore be the result of a pore block through insertion of (a part of) the molecule into the receptor pore. This pore block would then prevent influx of chloride ions following binding of GABA to the GABA binding site. Full elucidation of the precise mode of action of PFAS on α_1_β_2_γ_2L_ GABA_A_ receptor function would require extensive mechanistic research, including measuring voltage-current curves in presence and absence of PFAS. Such experiments may elucidate whether PFOS and/ or PFOA cause a voltage-dependent pore block, which is a likely mechanism of action given our results. However, regardless the exact molecular mechanism, the antagonistic effects of PFOS and PFOA on the human GABA_A_ receptor can be hypothesized to cause hyperexcitation in neuronal networks.

This hypothesis is further supported by evidence that PFOS induces the influx of extracellular calcium^39^. However, when primary rat cortical neurons are exposed to PFAS, excitation is observed only in spike and burst activity at 100 μM PFOS, whereas no significant excitatory effects of PFOA were found (Figs. 2 and 6). As there were no effects on primary rat cortical neurons at low or submicromolar levels of PFAS, as observed for human GABA_A_ receptor, two different hiPSC-derived neuronal co-cultures were exposed to PFOS and PFOA to exclude inter-species differences. In contrast to the expected hyperexcitation, neither of the hiPSC-derived neuronal models showed hyperexcitation following acute exposure to PFOS or PFOA. In the iCell Glutaneurons – iCell Astrocytes co-culture, a strong inhibition of activity in combination with a prolonged burst duration was found following exposure to PFOS or PFOA (Figs. 3, 4 and 6). This model showed spike and network burst LOECs for PFOS (0.1 µM) and PFOA (1 µM) that were comparable to the human GABA_A_ receptor studies. In contrast, in the SynFire iNS co-culture showed an apparent lack of sensitivity comparable to the rat primary cortical culture (Figs. 5, 6). While the human iPSC models thus differ in sensitivity, they both show inhibition of neuronal activity in contrast to the rat primary culture. This hints towards a specific species-difference. However, giving the inhibitory effect on the human α_1_β_2_γ_2L_ GABA_A_ receptor, these findings also imply that PFAS may have additional targets that mask the inhibition of the GABA_A_ receptor and/or that compensatory mechanisms are at play in neuronal networks to dampen the loss of GABA_A_ receptor function. For example, PFAS could act on different neurotransmitter receptors, such as glutamate receptors, thereby masking the inhibition of the GABA_A_ receptor. This idea is supported by research that showed that PFOS induces NMDA receptor mediated excitotoxicity^59^. Toxicity results of a high influx of extracellular Ca^2+^, which in its turn over-activates the NMDA receptor. Although this study supports the argument that PFAS hit multiple targets, it should be noted that these results were obtained following 24 h exposure, as compared to our acute exposure study. Alternatively, the hyperexcitability effects of PFOS and PFOA may become apparent only after chronic exposure. These effects may then be the result of changes in for example the AMPA receptor composition or expression, as it has been previously shown that prolonged exposure of rat primary cortical neurons to 1 µM PFOS lowers the expression of the AMPA receptor subunit GluR2, resulting in increased intracellular calcium concentrations and enhanced glutamate sensitivity^60^. It is also possible that the differences between the rodent and human neuronal model are due to an immature phenotype of the hiPSC-models. However, the used models have been shown to have a mature phenotype with hyperpolarizing GABA-responses and expression of mature transporters like KCC2 and NKCC^61,62^.

Compared to the two hiPCs-derived neuronal models, the rat primary cortical culture contains the highest ratio of astrocytes, about 45%^63,64^. Astrocytes increase the level of network activity^55,65,66^ and modulate responses to toxicological insults^67^. It has been shown that astrocytes can also provide protection against adverse effects of PFOS on rat hippocampal neurons^68^. Therefore, the lack of an excitatory effect at lower concentrations could be due to the protective effect of the astrocytes present. When astrocytes are exposed for 24 h to concentrations higher than 50 μM PFOS, astrotoxicity starts to occur, thereby increasing the risk on excitotoxicity^68^. Although exposure duration in our study was shorter and no cytotoxicity was observed, the increase in spike and burst activity following acute exposure of rat primary cortical neurons to a high PFOS concentration could be indicative of the onset of excitotoxicity. Another potential explanation for the inhibition of activity in the hiPSC-derived neuronal cultures could reside in the PFOS-induced shift in the activation curves of voltage-gated sodium channels towards hyperpolarization^37,69^, making it harder for the cell to reach an action potential. Future research to elucidate the possible counteracting mechanisms of action observed in our network study of PFOS and PFOA could focus on patch-clamp studies in neuronal networks following both acute and chronic exposure.

For PFOS and PFOA human serum to plasma ratios are 1:1^70^. Reported plasma or serum concentrations for PFOA fall for the general population in the range between 0.001–0.2 µM^71–73^. In the occupationally exposed population, levels as high as 2.5 µM are found^70^. Thus, LOECs reported on GABA_A_ receptor function, spike rate and network burst activity (1 µM) are below this level. Importantly, PFOA can cross the blood-brain barrier^72^. Therefore, PFOA could be a risk for the occupationally exposed population. PFOS can cross the blood-brain barrier less efficient than PFOA^72^, but the LOECs reported here for PFOS are lower than for PFOA. Reported plasma or serum concentrations for PFOS fall in the range between 0.002–1.3 µM in the general population^71–73^ and in occupationally exposed workers the levels are even higher, ranging from 0.27 to 10 µM^70,71^. LOECs on GABA_A_ receptor data and on spike activity in an hiPSC-derived neuronal co-culture reported in this study are within or below the range found in human plasma levels. It should be noted though that human exposure to PFAS is low dose, but continuous. Our *in vitro* study should therefore be considered mainly as hazard characterisation, highlighting the potential target(s) of PFOS and PFOA. Our findings, combined with the fact that PFAS can cross the blood-brain barrier, indicate that these compounds have a clear potential to cause neurotoxic effects in the occupationally exposed populations as well as in the general population.

## Material and Methods

### Animals

All experiments were conducted in accordance with Dutch law and approved by the Ethical Committee for Animal Experiments of Utrecht University. Animals were treated humanely and all efforts were made to alleviate suffering. *Xenopus laevis* oocytes were purchased from EcoCyte Bioscience (Castrop-Rauxel, Germany). Primary cultures of rat cortical neurons were prepared from postnatal day (PND) 0–1 Wistar rat pups (Envigo, Horst, The Netherlands).

### Chemicals

MgSO_4_, NaHCO_3_ and Ca(NO_3_) were purchased from Merck (Darmstadt, Germany). Neurobasal-A medium, Dulbecco’s Modified Eagle Medium (DMEM)-F12, penicillin – streptomycin (5000 U/mL – 5000 µg/mL for rat primary cortical culture media and 10.000 U/mL – 10.000 µg/mL for hiPSC medium), B27 plus supplement, N2 supplement and l-glutamine were obtained from Life Technologies (Bleiswijk, The Netherlands). iCell Neural Supplement B and Nervous System Supplement were provided by Cellular Dynamics International (Madison, WI, USA). BrainPhys neuronal medium was obtained from StemCell Technologies (Cologne, Germany). SynFire seeding basal medium, short-term basal medium, long-term basal medium and accessory supplements were provided by NeuCyte (Sunnyvale, CA, USA). Laminin (L2020), 50% polyethyleneimine (PEI) solution, sodium borate, boric acid and all other chemicals (unless stated otherwise) were obtained from Sigma-Aldrich (Zwijndrecht, The Netherlands).

Stock solutions of PFOS (>98% purity, Fluka, Zwijndrecht, The Netherlands) and PFOA (>96% purity, Acros Organics, Geel, Belgium) were made in purity-checked dimethyl sulfoxide (DMSO) and stored at 4 °C. Final concentration of DMSO in PFOS- or PFOA-containing solutions was always kept below 0.1% (vol/vol). DMSO at concentrations up to 0.5% (vol/vol) had no effect on GABA_A_ receptor-mediated currents.

### α_1_β_2_γ_2L_ GABA_A_ receptor expression in Xenopus laevis oocytes

All procedures have been described previously^43,48^. Briefly, complementary DNA (cDNA) coding for the human α_1_, β_2_ (Origene, Rockville, USA) and human γ_2L_ subunits (kindly provided by Dr Paul J. Whiting, Merck Sharp & Dohme Research Laboratories, Neuroscience Research Center, Harlow, Essex, U.K.) was dissolved in distilled water at a 1:1:1 molar ratio and injected (23 nL/oocyte, ~1 ng of each subunit) into the nuclei of stage V or VI oocytes using a Nanoject Automatic Oocyte Injector (Drummond, Broomall, PA). Following injection, oocytes were incubated at 21 °C in modified Barth’s solution containing (in mM) 88 NaCl, 1 KCl, 2.4 NaHCO_3_, 0.3 Ca(NO_3_)_2_, 0.41 CaCl_2_, 0.82 MgSO_4_, 15 HEPES, and 10 μg/mL neomycin (pH 7.6 with NaOH).

### Electrophysiological recordings of GABA_A_ receptor currents

Electrophysiological recordings were performed on oocytes following 4–6 days of incubation to ensure sufficient translation of injected cDNA and functional expression of α_1_β_2_γ_2L_ GABA_A_ receptors in the membrane. Ion currents associated with GABA_A_ receptor activity were measured with the two-electrode voltage-clamp technique using a Gene Clamp 500B amplifier (Axon Instruments, Union City, CA) with high-voltage output stage as described previously ^43,48^. Recording microelectrodes (0.1–1 MΩ) were filled with KCl (3 M). Oocytes, placed in a custom-built Teflon oocyte recording chamber, were voltage-clamped at −60 mV and continuously superfused (~30 mL/min) with saline solution containing (in mM) 1 CaCl_2_, 10 HEPES (pH 7.2 with NaOH), 2.5 KCl, 115 NaCl. Membrane currents were low-pass filtered (8-pole Bessel; 3 dB at 0.3 kHz), digitized (12 bits; 1024 samples per record), and recorded on disk using WinWCP (Strathclyde Electrophysiological Software, Glasgow, Scotland) for further analysis.

Aliquots of freshly thawed GABA stock solution in distilled water and PFOS or PFOA in DMSO were added to the saline immediately before the experiments. GABA-responsive oocytes were exposed to saline, saline containing GABA (34 µM or 1 mM), saline containing a PFAS or saline containing GABA in combination with a PFAS by switching the perfusate using a servomotor-operated valve. To determine whether oocytes were GABA responsive, oocytes were super-fused for 40 s with a saline solution containing GABA. If oocytes were responsive, they were exposed for 10 s to saline containing GABA, followed directly by a 20 s exposure of saline containing GABA and PFAS and finally to 10 s of saline containing GABA. Concentrations of 0.01–100 µM PFOS or PFOA were chosen to overlap with earlier *in vitro* studies and extended to include human serum and plasma concentrations. A washout period of 4 min between each application was introduced, allowing receptors to recover from desensitization. Sham-injected oocytes did not show any ion current upon superfusion with GABA, PFOS, or PFOA at concentrations tested, alone or in co-application with GABA (data not shown). To minimize adsorption of PFOS or PFOA to the perfusion system, glass reservoirs and Teflon tubes (polytetrafluoroethylene, Rubber, Hilversum, The Netherlands) were used.

### Cell culture

Primary rat cortical cultures and hiPSC-derived neuronal co-cultures were kept at 37 °C in a humidified 5% CO_2_ incubator. All cell culture surface materials were pre-coated with 0.1% PEI solution diluted in borate buffer (24 mM sodium borate/50 mM boric acid in Milli-Q adjusted to pH 8.4).

Primary rat cortical cells were isolated from PND0–1 Wistar rat pups as described previously^50,64^ with minor modifications. Briefly, PND0–1 pups were decapitated and cortices were rapidly dissected on ice and kept in serum free dissection medium (Neurobasal-A supplemented with 25 g/L sucrose, 450 µM l-glutamine, 30 µM glutamate, 1% penicillin/streptomycin and 2% B27 plus supplement, pH 7.4) during the entire procedure. Cortices were dissociated to a single-cell suspension by mincing with scissors, trituration and filtering through a 100 µm mesh (EASYstrainer, Greiner). The cell suspension was diluted to a 2 × 10^6^ cells/mL solution. Droplets of 50 µL were placed on the electrode fields in wells of pre-coated 48-well MEA plates (Axion BioSystems Inc., Atlanta, GA, USA). Cells were left to adhere for ~2 hr before adding 450 µL serum free dissection medium. At DIV4, 90% of the serum free dissection medium was replaced with serum free culture medium (Neurobasal-A supplemented with 25 g/L sucrose, 450 µM l-glutamine, 1% penicillin/streptomycin and 2% B27 plus supplement, pH 7.4). Rat primary cortical neurons were exposed at DIV9–11.

iCell Glutaneurons (Lot# 103288, containing ~70% glutamatergic and ~30% GABAergic neurons); Cellular Dynamics International, Madison, WI, USA) and iCell Astrocytes (Lot# 103956; Cellular Dynamics International, Madison, WI, USA) were thawed and cultured according to manufacturer’s protocol. In short, each cell type was thawed separately in supplemented BrainPhys medium (BrainPhys medium supplemented with 2% iCell Neural Supplement B, 1% Nervous System Supplement, 1% N2, 1% penicillin – streptomycin and 0.1% laminin). Following centrifugation, the cell pellet was diluted in dotting medium (supplemented BrainPhys medium with 10% laminin) till 15 × 10^3^ cells/µL for iCell Glutaneurons and 6.7 × 10^3^ cells/µL for iCell Astrocytes. Before plating, iCell Glutaneurons and iCell Astrocytes were premixed into a co-culture containing 120 × 10^3^ iCell Glutaneurons and 20 × 10^3^ iCell Astrocytes. Cells were plated in 11 µL droplets (140 × 10^3^ cells/droplet with 85% iCell Glutaneurons and 15% iCell Astrocytes) over the electrode field of pre-coated 48-well MEA plates. After plating, cells were allowed to adhere for ~1 hr following which 300 µL of room temperature (RT) supplemented BrainPhys medium was added. 50% Medium changes with RT supplemented BrainPhys medium took place at DIV1, 2, 4, 6, 8, 10, 12 and 14. The iCell Glutaneurons – iCell Astrocytes cultures were exposed at DIV14.

SynFire glutamatergic neurons (Lot# 118B and 000124), SynFire GABAergic neurons (Lot# 118B and 000124) and SynFire astrocytes (Lot# 13029; all from NeuCyte Sunnyvalle, CA, USA) were thawed and cultured according to manufacturer’s protocol. Briefly, each cell type was thawed separately in DMEM-F12. Each cell pellet was dissolved in complete seeding medium (containing the seeding supplement) till a density of 10 × 10^3^ cells/µL following which a mixture was made containing 52% glutamatergic neurons, 22% GABAergic neurons and 26% astrocytes. This mixture was plated in 50 µL droplets (270 × 10^3^ cells per droplet) over the electrode field of pre-coated MEA plates. Cells were placed in the incubator and left overnight to adhere. The next day, 250 µL/ well of complete short-term maintenance medium (containing the supplement) was added. At DIV3 and 5, 50% medium changes with short-term maintenance medium took place, after which culture medium was gradually replaced by complete long-term maintenance medium (containing supplements A and B) through 50% medium changes at DIV7, 10, 13, 16, 19, 22, 25 and 25. SynFire iNS co-cultures were exposed at DIV28.

### MEA measurements

Each well of a 48-well MEA plate contains 16 nanotextured gold micro-electrodes (~40–50 µm diameter; 350 µm spacing) with 4 integrated ground electrodes yielding a total of 768 channels for simultaneous recording (for review see^74^). Spontaneous electrical activity was recorded as described previously^52,64^. Briefly, signals were recorded at the day of experiments (DIV9–11 for rat primary cortical cultures, DIV14 for iCell Glutaneurons – iCell Astrocytes co-cultures and DIV28 for SynFire iNS co-cultures) using a Maestro 768-channel amplifier with integrated heating system and temperature controller and a data acquisition interface (Axion BioSystems Inc., Atlanta, GA, USA). Data acquisition was managed with Axion’s Integrated Studio (AxIS 2.4.2.13) and recorded as.RAW files. All channels were sampled simultaneously with a gain of 1200x and a sampling frequency of 12.5 kHz/channel with a 200–5000 Hz band-pass filter. Prior to the recording, MEA plates were allowed to equilibrate for 5–10 min in the Maestro.

In order to determine effect of reference compounds on spontaneous neuronal activity (spiking and (network) bursting behaviour) on the three different cell cultures, a 30 min baseline recording was made. Following this baseline recording, wells were exposed (10 × dilution for primary rat cortical cultures and SynFire iNS co-culture and 30 × dilution for iCell Glutaneurons – iCell Astrocytes co-culture) to PFOS, PFOA or the solvent control and directly a 30 min exposure recording was made. In order to overlap with earlier *in vitro* experiments and to fall in the range of reported human serum and plasma levels of PFOS and PFOA, concentrations of 0.1–100 µM were tested. Stock solutions of PFOS and PFOA in DMSO were diluted in culture medium to obtain desired concentrations. In all experiments, the solvent concentration never exceeded 0.1% v/v. In order to prevent receptor (de)sensitization each well was exposed to a single concentration. For each experimental condition, MEA plates from at least two different plating rounds were used.

### Data analysis and statistics

Peak amplitudes of GABA-evoked ion currents were measured and normalized to the maximal amplitude (at 1 mM) of GABA-evoked control responses to adjust for differences in receptor expression levels among oocytes and for small variations in response amplitudes over time as described previously ^43,48^. Normalized ion currents were plotted against GABA concentration in each experiment. GABA concentration-effect curves were fitted to the data obtained in separate experiments using Prism (Graphpad Software, La Jolla, CA, USA). The percentage of PFOS- or PFOA-induced inhibition of the GABA-evoked ion current was calculated from the quotient of the maximum amplitude of the GABA-PFOS/PFOA co-application response and the maximum amplitude of the control (GABA) response.

To determine (modulation of) spontaneous activity,.RAW data files were re-recorded to obtain Alpha Map files. In this re-recording, spikes were detected with the AxIS spike detector (Adaptive threshold crossing, Ada BandFIt v2) using a variable threshold spike detector set at 7× (rat primary cortical cultures) or 5.5× (hiPSC cultures) standard deviation (SD) of internal noise level (rms) on each electrode. Post/pre-spike duration was set to 3.6/2.4 ms respectively. For further data analysis, spike files were loaded in NeuralMetric Tool (version 2.2.4, Axion BioSystems) and only active electrodes (MSR ≥ 6 spikes/min) in active wells (≥1 active electrode) were included. Since (network) bursting behaviour is crucial for *in vivo* neuronal communication^75^, it was analysed using the Poisson Surprise method^76^ with a minimal surprise of 10 and a minimum bursting frequency of 0.005 bursts/sec. An adaptive threshold algorithm was used for the extraction of network bursts.

The effects of PFOS and PFOA on spontaneous neuronal activity were determined by comparing the baseline activity with activity following exposure. A custom-made MS Excel macro was used to calculate treatment ratios (TR) per well for the different metric parameters (Table 3) by: (parameter_exposure_/parameter_baseline_) × 100%. Hereafter, TRs were normalized to appropriate vehicle control (DMSO). To prevent inclusion of exposure artefacts, effect analysis was performed 20–30 min post-exposure.

Oocytes or wells that showed effect two times SD above or below average were considered outliers and removed for further data analysis (1.1% for oocytes, 4.2% for rat primary cortical neurons, 4.7% for iCell Glutaneuron – iCell Astrocytes co-cultures and 3.6% for SynFire iNS co-cultures). For oocyte data, the concentration-dependent effects of PFOS and PFOA were determined by one-way ANOVA and a post hoc Bonferroni test. Best-fit concentration-response curves were plotted in PRISM (version 8.0.1) by nonlinear regression with a four-parameter variable slope with the bottom value constrained to 0 and top value to 100 to improve the fit. In the case of MEA data, concentration-dependent effects were determined by a one-way Welch ANOVA and a post hoc Dunnett test. *P-*values < 0.05 were considered statistically significant. All statistical analyses were performed in R version 3.6.0 (R core team 2019) with base R or by using the DescTools package (by Signorell *et al*., 2019, version 0.99.28).

Data are presented as mean ± standard error of the mean (SEM) from the number of oocytes or wells (*n*) indicated, derived from 1 to 2 independent batches (*N)* of oocytes, 2 plating rounds (*N*) for the rat primary cortical culture, 5 plating rounds (*N*) for the iCell Glutaneuron – iCell Astrocytes co-culture and 2 independent plating rounds (*N*) for the SynFire iNS co-culture unless stated otherwise. IC_50_ values are reported with confidence intervals (CI). LOECs are defined as lowest statistically significant concentrations.

## Supplementary information

Supplementary information.

## Data Availability

The datasets generated during and/or analysed during the current study are available from the corresponding author on reasonable request.
