# Supplementary material for: Perfluorooctane sulfonate (PFOS) and perfluorooctanoate (PFOA) acutely affect human α1β2γ2L GABAA receptor and spontaneous neuronal network function in vitro
Source: Sci Rep. 2020 Mar 24;10:5311. doi: 10.1038/s41598-020-62152-2 (PMC7093421; doi:10.1038/s41598-020-62152-2)
Supplement: Supplementary file 1 — Supplementary information. [file 41598_2020_62152_MOESM1_ESM.pdf]

Perfluorooctane sulfonate (PFOS) and perfluorooctanoate (PFOA) acutely affect human $\alpha_1\beta_2\gamma_{2L}$ $GABA_A$ receptor and spontaneous neuronal network function *in vitro*

Anke M. Tukker, Lianne M.S. Bouwman, Regina G.D.M. van Kleef, Hester S. Hendriks, Juliette Legler and Remco H.S. Westerink

Supplemental data

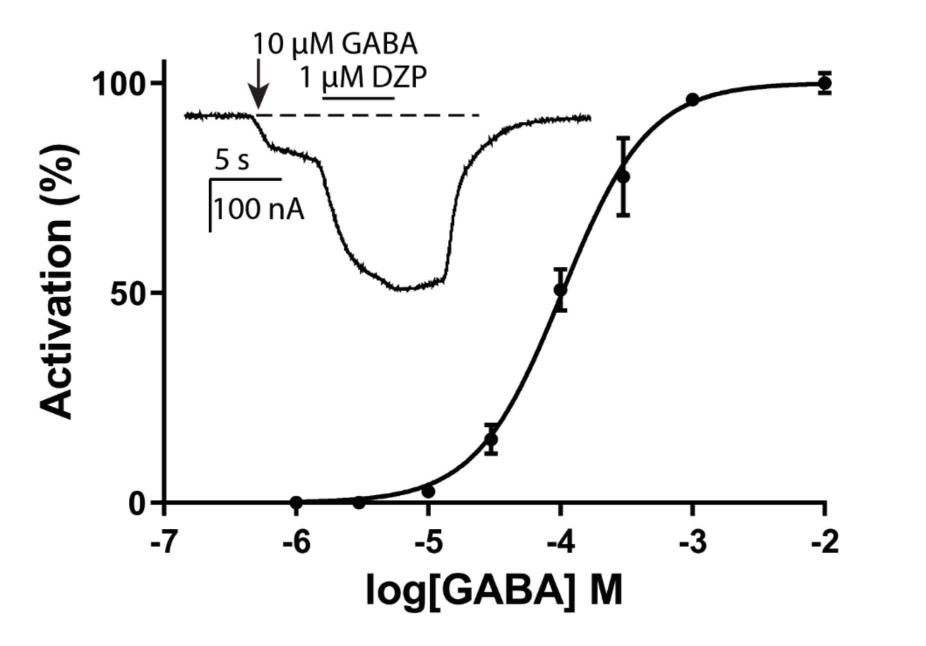

Fig S1 Concentration-effect curve of the GABA-evoked ion current in oocytes expressing the $\alpha_1\beta_2\gamma_{2L}$ subunits forming the $GABA_A$ receptor. Curve is fitted based on mean values (± SD) from 4 – 16 oocytes. Co-exposure of GABA and 1 µM diazepam (DZP) evokes a potentiation confirming the presence of a functional γ_{2L} subunit (insert).

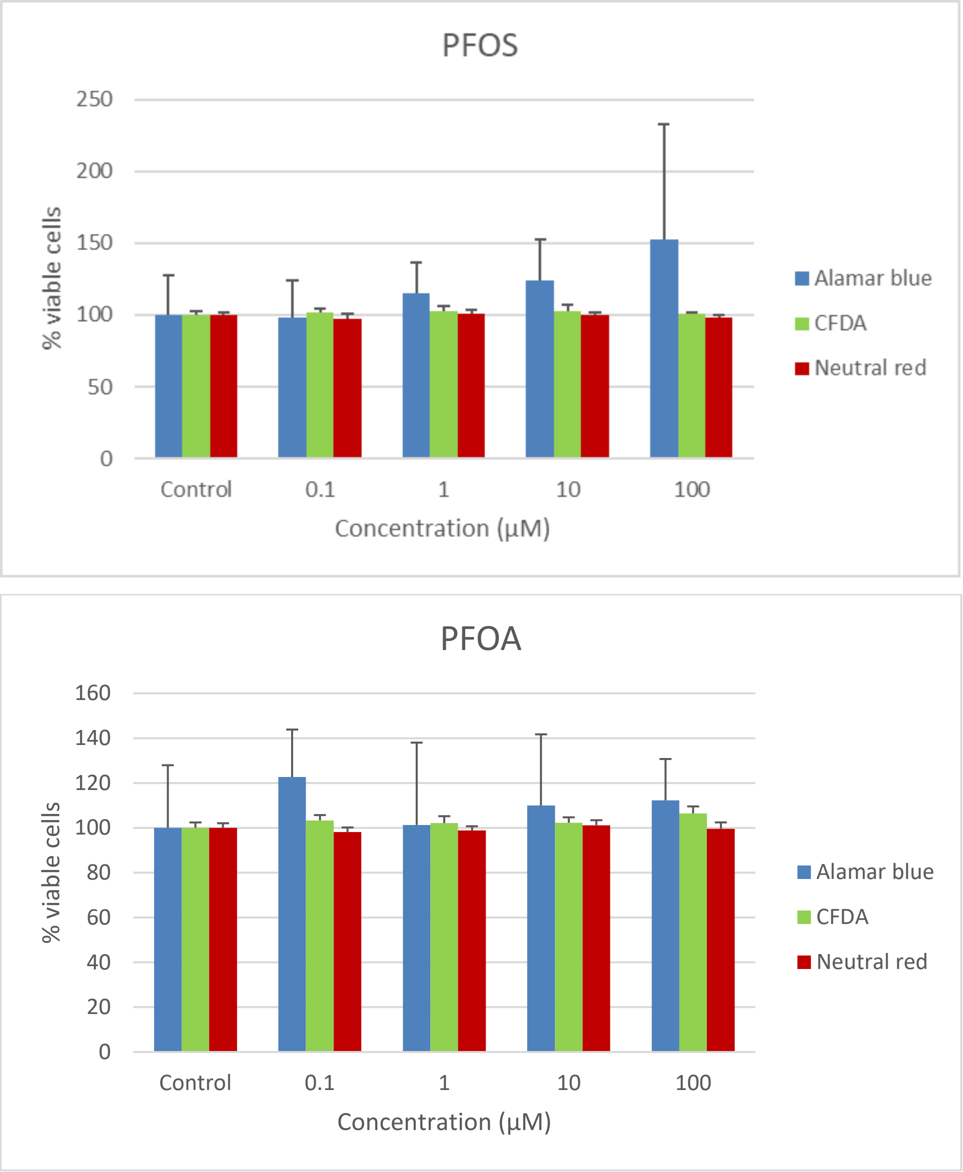

Fig S2 Cytotoxicity data of PFOS (top) and PFOA (bottom) from an alamar blue (blue), CFDA (green) and neutral red (red) assay measured 24 h after a 30 min exposure of rat primary cortical cultures. No cytotoxicity can be observed. *n* = 6 – 12 wells, N = 2 plates. Data is presented as mean ± SD.
